# VARC-3 defined outcome of valve-in-valve transcatheter aortic valve implantation in stentless compared with stented aortic bioprostheses

**DOI:** 10.1007/s00392-023-02347-5

**Published:** 2023-12-11

**Authors:** Jean-Honoré Steul, Mohamed Abdel-Wahab, Tomasz Stankowski, Stephan Haussig, Felix J. Woitek, Tomasz Gasior, Lisa Crusius, Luise Knorr, Felicitas V. Müller, Dirk Fritzsche, Philipp Kiefer, Utz Kappert, David Holzhey, Axel Linke, Norman Mangner

**Affiliations:** 1 Department of Internal Medicine and Cardiology, Heart Center Dresden, Technische Universität Dresden, Fetscherstr. 76, 01307 Dresden, Germany; 2 Department of Cardiology, Heart Center Leipzig at University of Leipzig, Leipzig, Germany; 3 Department of Cardiac Surgery, Sana Heart Center Cottbus, Cottbus, Germany; 4 Boehringer Ingelheim International GmbH, Ingelheim, Germany; 5 Department of Cardiac Surgery, Heart Center Leipzig at University of Leipzig, Leipzig, Germany; 6 Department of Cardiac Surgery, Heart Center Dresden, Technische Universität Dresden, Dresden, Germany; 7 Department of Cardiac Surgery, Helios University Hospital Wuppertal, Wuppertal, Germany

**Keywords:** Valve-in-valve, Transcatheter aortic valve implantation, Stentless bioprosthesis, Stented bioprosthesis, VARC-3

## Abstract

**Background:**

Valve-in-valve (ViV) transcatheter aortic valve implantation (TAVI) is a viable alternative to redo surgery in selected patients with bioprosthetic valve dysfunction. Most ViV-TAVI procedures have been performed in stented bioprosthetic valves (ST); stentless bioprostheses (SL) lack fluoroscopic markers and could be more challenging for ViV-TAVI. Data on more recent patients applying Valve Academic Research Consortium (VARC)-3 defined outcomes are scarce. We compared patient characteristics, procedural outcomes, and 5-year mortality of patients with SL versus ST aortic bioprosthetic valve failure undergoing ViV-TAVI.

**Methods:**

Patients undergoing ViV-TAVI between 2007 and 2022 (52.5% of cases after 2015) at 3 German centers were included in this analysis. The co-primary outcome measures were technical success, device success, and early safety defined by VARC-3. Mortality was assessed up to 5 years.

**Results:**

Overall, 43 (11.8%) SL and 313 (88.2%) ST ViV-TAVI were included. Patients were comparable with regard to age, sex, clinically relevant baseline comorbidities, and surgical risk.

Technical success (SL: 83.7% versus ST: 79.9%, *p* = 0.552), device success (SL: 67.4% versus ST: 54.3%, *p* = 0.105), and early safety (SL: 74.4% versus ST: 66.5%, *p* = 0.296) were comparable between groups. The 30-day mortality (SL: 7.0% versus ST: 2.6%, *p* = 0.136) and 5-year mortality rates (SL: 23.3% versus ST: 24.6%, *p* = 0.874) were not significantly different between groups.

**Conclusion:**

SL and ST ViV-TAVI led to comparable short-term outcomes according to VARC-3- defined endpoints and similar mortality rates up to 5 years of follow-up.

**Graphical abstract:**

**Figure:**
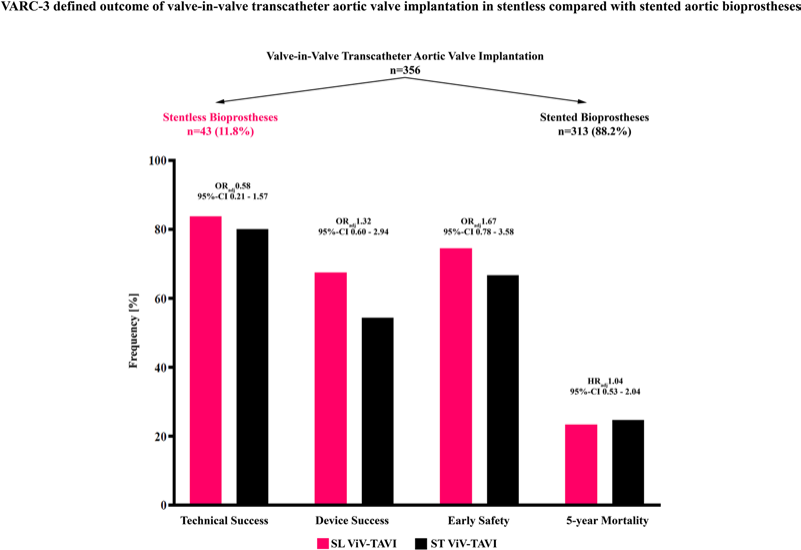
VARC-3 defined technical success, device success, and early safety as well as 5-year all-cause mortality in patients undergoing valve-in-valve transcatheter aortic valve implantation (ViV-TAVI) into stentless (SL) compared with stented (ST) failed aortic bioprostheses.

**Supplementary Information:**

The online version contains supplementary material available at 10.1007/s00392-023-02347-5.

## Introduction

Bioprosthetic valves have been increasingly used for surgical aortic valve implantation over the last two decades [1, 2]. The obvious benefit of bioprosthetic over mechanical valves is no need for permanent oral anticoagulation. However, a major disadvantage of bioprostheses is a higher rate of structural valve deterioration and failure, thus frequently leading to reintervention after 10–20 years [3]. Bioprosthetic valves can be divided into stented and stentless prostheses. Stented valves are composed of valve leaflets that are attached to a stent frame and a circular or scallop-shaped external sewing ring, whereas stentless valves have neither a stent frame that supports valve leaflets nor a base ring [3]. A majority of the patients nowadays is treated with stented bioprostheses, but roughly 10% of the patients undergoing bioprosthetic aortic valve replacement receive a stentless valve [4].

Compared to primary operation, re-operation for a failing aortic bioprosthesis is associated with increased mortality and morbidity, in particular in elderly and comorbid patients [5, 6]. Valve-in-valve (ViV) transcatheter aortic valve implantation (TAVI) has become an important treatment strategy in selected patients with bioprosthetic valve dysfunction [7, 8] and its application has increased over the last decade [9]. Most ViV-TAVI procedures worldwide have been performed in failing stented bioprosthetic valves [10]; however, ViV-TAVI in stentless compared with stented valves appears to be more challenging due to the lack of fluoroscopic markers and is associated with a higher rate of periprocedural complications [10]. So far, Duncan et al. examined the largest cohort of ViV-TAVI comparing stentless with stented bioprostheses; however, the group comprised only patients treated between 2007 and May 2016 lacking contemporary ones treated with the latest device iterations. Moreover, the latest version of the Valve Academic Research Consortium (VARC-3) provides updated definitions for procedural success and safety [11].

Against this background, we aimed to compare the baseline characteristics, procedural outcomes as well as the mortality up to 5 years in a more contemporary cohort of patients receiving ViV-TAVI in stentless versus stented surgical bioprostheses applying VARC-3 definitions.

## Methods

### Study population

All consecutive patients undergoing ViV-TAVI for degenerated stented and stentless surgical aortic bioprostheses at three German tertiary heart centers were enrolled in a multicenter registry. The treatment period was from 09/2007 to 07/2022. The study complies with the declaration of Helsinki and data acquisition and follow-up examinations were approved by each local ethic committee. All patients provided written informed consent before the procedure.

### Data collection

Baseline characteristics, procedural data, and outcome data were prospectively collected and follow-up was performed at 30 days post-procedure and afterward every 12 months at ambulatory visits or by phone. True inner diameter of the valves was determined as published [12]. Mode of failure was classified as stenosis, regurgitation or ‘mixed’ if regurgitation ≥ grade 2 (on a scale of 3) was present in a predominantly stenotic valve. Pre-existing prosthesis–patient mismatch (PPM) was estimated according to patient body size and bioprosthetic valve type and size in the VIVID calculator and graded as none/mild, moderate, or severe according to the indexed effective orifice area > 0.85 cm^2^/m^2^, 0.85–0.66 cm^2^/m^2^, and ≤ 0.65 cm^2^/m^2^ in patients with a body mass index < 30 kg/m^2^ and > 0.70 cm^2^/m^2^, 0.70–0.56 cm^2^/m^2^, and ≤ 0.55 cm^2^/m^2^ in patients with a body mass index ≥ 30 kg/m^2^, respectively. Presence of lung disease, immunosuppressant medication, diabetes mellitus, coronary heart disease (CAD), cerebrovascular disease, and peripheral artery disease were defined according to the STS-PROM definitions [13].

### Outcome measures

All clinical outcomes were defined according to the updated definitions of the Valve Academic Research Consortium (VARC-3) [11]. The co-primary outcome measures were technical success, device success, and early safety as defined by VARC-3 [11]. Briefly, technical success is a composite of freedom from mortality; successful access, delivery of the device, and retrieval of the delivery system; correct positioning of a single prosthetic heart valve into the proper anatomical location; and freedom from surgery or intervention related to the device or to a major vascular or access-related, or cardiac structural complication at the exit of the operation room. Device success is a composite of technical success, freedom from mortality; freedom from surgery or intervention related to the device or to a major vascular or access-related or cardiac structural complication; and intended performance of the valve (mean gradient < 20 mmHg, peak velocity < 3 m/s, Doppler velocity index ≥ 0.25, and less than moderate aortic regurgitation) at 30 days. Early safety is a composite of freedom from all-cause mortality; freedom from all stroke; freedom from VARC type 2–4 bleeding; freedom from major vascular, access-related, or cardiac structural complication; freedom from acute kidney injury stage 3 or 4; freedom from moderate or severe aortic regurgitation; freedom from new permanent pacemaker due to procedure-related conduction abnormalities; and freedom from surgery or intervention related to the device at 30 days.

Secondary outcome measures included the individual components of the aforementioned composite outcomes as well as 30-day, 1-year, 3-year, and 5-year all-cause mortality.

### Statistical analysis

Data are presented as numbers and frequencies for categorical and as median (interquartile range, IQR) for continuous variables. Categorical variables were compared using the *χ*2 test or Fisher’s exact test as appropriate. Continuous variables were compared using the Mann–Whitney *U* test after testing for variable distribution applying the Shapiro–Wilk test.

Predictors of the primary outcome measures technical success, device success, and early safety were evaluated with a binary logistic regression analysis. Clinically relevant baseline variables with a *p*-value ≤ 0.1 in univariate analysis were included after excluding collinearity. Age, sex, and stentless versus stented surgical bioprostheses were forced into the models.

Estimates of 1-year, 3-year, and 5-year all-cause mortality were analyzed according to the method of Kaplan–Meier and group comparisons were made applying the log-rank test. Independent predictors of 5-year all-cause mortality were determined with a Cox proportional hazard regression model. Clinically relevant baseline variables with a *p*-value ≤ 0.1 in univariate analysis were included after excluding collinearity. Age, sex, and stentless versus stented surgical bioprostheses were forced into the models.

Collinearity was assumed if R was greater than 0.70 in the bivariate correlation test, the tolerance value was below 0.10, and/or the variable inflation factor (VIF) was greater than 10. Missing values were not imputed in the model.

To account for changes over time, a sensitivity analysis for characteristics and outcomes according to two treatment periods (2007–2015 and 2016–2022) was performed.

The statistical analysis was performed using SPSS Statistics version 27.0 (IBM Corporation, Armonk, USA). A *p*-value < 0.05 was considered as statistically significant.

## Results

### Baseline and procedural characteristics

Out of 356 VIV-TAVI cases, 43 (11.8%) and 313 (88.2%) patients had a pre-existing stentless (SL) or stented (ST) surgical bioprosthesis, respectively (Online Table I). ViV-TAVI was performed between 09/2007 and 07/2022 with 52.5% of the cases performed after 2015. Baseline characteristics are shown in Table 1. They were balanced between groups with regard to age, sex, perioperative risk, and most baseline comorbidities except higher rates of previous PCI (*p* = 0.032), lower rates of chronic obstructive pulmonary disease (*p* = 0.032), and a lower left ventricular ejection fraction (LV-EF) (*p* = 0.032) in SL compared with ST.

**Table 1:** Baseline characteristics according to valve type

	Total ViV cohort (*n* = 356)	Stentless valve (*n* = 43)	Stented valve (*n* = 313)	*p*-value
Age [years]	79 (75; 82)	76 (74; 82)	79 (75; 83)	0.084
Male sex, *n* (%)	207 (58.1)	30 (69.8)	177 (56.5)	0.099
Body mass index [kg/m^2^]	27.3 (24.2; 30.8)	26.1 (22.4; 30.8)	27.4 (24.4; 30.8)	0.226
STS-PROM [%]	7.0 (4.6; 11.4)	7.7 (4.8; 12.5)	7.0 (4.5; 11.3)	0.616
NYHA III/IV, *n* (%)	282 (79.2)	36/43 (83.7)	246/313 (78.6)	0.437
Coronary artery disease, *n* (%)	199/356 (55.9)	24/43 (55.8)	175/313 (55.9)	0.990
Previous myocardial infarction, *n* (%)	41/356 (11.5)	6/43 (14.0)	35/313 (11.2)	0.593
Previous PCI, *n* (%)	72/356 (20.2)	14/43 (32.6)	58 (18.5)	0.032
Previous CABG, *n* (%)	124/356 (34.8)	10/43 (23.3)	114/313 (36.4)	0.089
Atrial fibrillation, *n* (%)	140/356 (39.3)	14/43 (32.6)	126/313 (40.3)	0.333
Hypertension, *n* (%)	340/356 (95.5)	40/43 (93.0)	300/313 (95.8)	0.424
Diabetes mellitus, *n* (%)	130/356 (36.5)	16/43 (37.2)	114/313 (36.4)	0.920
Previous stroke, *n* (%)	45/356 (12.6)	9/43 (20.9)	36/313 (11.5)	0.081
PAD, n (%)	62/356 (17.4)	8/43 (18.6)	54/313 (17.3)	0.826
Carotid stenosis, *n* (%)	61/355 (17.2)	9/43 (20.9)	52/312 (16.7)	0.487
COPD, *n* (%)	117/354 (33.1)	8/43 (18.6)	109/311 (35.0)	0.032
CKD > 3b, *n* (%)	125/356 (35.1)	13/43 (30.2)	112/313 (35.8)	0.475
Immunocompromised status, *n* (%)	21/356 (5.9)	3/43 (7.0)	18/313 (5.8)	0.729
Pacemaker prior to ViV, *n* (%)	68/356 (19.1)	9/43 (20.9)	59/313 (18.8)	0.745
Left Ventricular Ejection Fraction [%]	55 (45; 60)	50 (45; 60)	55 (48; 61)	0.032
Mean gradient [mmHg]	36 (28; 45)	29 (17; 50)	37 (29; 45)	0.026
Time aortic valve replacement to ViV [years]	9.1 (6.6; 12.0)	10.5 (8.6; 13.0)	9 (6.4; 12.0)	0.011
Mode of Failure				< 0.001
Aortic stenosis, *n* (%)	194/356 (54.5)	17/43 (39.5)	177/313 (56.5)	
Aortic regurgitation, *n* (%)	39/356 (11.0)	14/43 (32.6)	25/313 (8.0)	
Mixed (Aortic stenosis + regurgitation ≥ 2), *n* (%)	123/356 (34.6)	12/43 (27.9)	111/313 (35.5)	
True internal diameter (TID)				< 0.001
TID < 20 mm, *n* (%)	167/342 (48.8)	0/37 (0)	167/305 (54.8)	
TID 20.0–22.99 mm, *n* (%)	104/342 (30.4)	6/37 (16.2)	98/305 (32.1)	
TID ≥ 23 mm, *n* (%)	71/342 (20.8)	31/37 (83.8)	40/305 (13.1)	
Pre-existing moderate/severe PPM, n (%)	171/318 (53.8)	7/33 (21.2)	164/285 (57.5)	< 0.001

Values are *n* (%) or median (IQR). ViV indicates valve-in-valve; *STS-PROM* Society of Thoracic Surgeons Predicted Risk of Mortality; *NYHA* New York Heart Association; *PCI* percutaneous coronary intervention; *CABG* coronary artery bypass graft; *PAD* peripheral artery disease; *COPD* chronic obstructive pulmonary disease; *CKD* chronic kidney disease; *TID* true internal diameter; *PPM* prosthesis–patient mismatch

The median time from aortic valve replacement to ViV-TAVI was significantly longer in SL compared with ST (10.5 (8.6; 13.0) years versus 9 (6.4; 12.0) years, *p* = 0.011). The mode of failure was restenosis, pure aortic regurgitation, and mixed disease (restenosis + regurgitation ≥ 2) in 39.5%, 32.6%, and 27.9% in SL, whereas those rates were 56.5%, 8.0%, and 35.5% in ST (*p* < 0.001). Surgical bioprostheses with a true internal diameter (TID) of < 20 mm were solely found in ST, whereas the rate of bioprostheses with a TID of ≥ 23 mm was highest in SL (*p* < 0.001). The rate of a pre-existing moderate/severe patient–prosthesis mismatch was significantly lower in SL compared with ST (*p* < 0.001).

Procedural details are outlined in Table 2 with no significant differences with regard to the access site and the use of self-expanding versus balloon-expandable TAVI devices. However, small differences were observed between specific TAVI devices and their different generations. In line with the higher rate of bigger pre-existing bioprostheses, larger TAVI devices were implanted in SL compared to ST. Four patients (all in ST) were in need for a second TAVI due to embolization in 2 cases (CoreValve, Sapien XT), one low implant causing severe aortic regurgitation (CoreValve), and one damaged delivery system after successful retrieval (EvolutR). The residual mean gradient was lower in SL compared with ST as was the rate of patients with a residual mean gradient ≥ 20 mmHg. The overall rates of moderate paravalvular aortic regurgitation were low but numerically higher in SL versus ST without reaching statistical significance. There was no severe paravalvular aortic regurgitation.

**Table 2:** Procedural characteristics and in-hospital hemodynamic outcomes

	Total ViV cohort (*n* = 356)	Stentless valve (*n* = 43)	Stented valve (*n* = 313)	*p*-value
Access				1.000
C 1Transfemoral, *n* (%)	335/356 (94.1)	41/43 (95.3)	294/313 (93.9)	
Transapical, *n* (%)	21/356 (5.9)	2/43 (4.7)	19/213 (6.1)	
Implanted THV				0.142
Self-expanding	275/356 (77.2)	37/43 (86.0)	240/313 (75.0)	
Balloon-expandable	81/356 (22.8)	6/43 (14.0)	75/313 (24.0)	
Specific THV				0.033
CoreValve	85/365 (23.9)	10/43 (23.3)	75/313 (24.0)	
EvolutR	185/365 (52.0)	26/43 (60.5)	159/313 (50.8)	
Portico	4/365 (1.1)	0/43 (0)	4/313 (1.3)	
Lotus	1/365 (0.3)	1/43 (2.3)	0/313 (0)	
Sapien XT	38/365 (10.6)	2/43 (4.7)	36/313 (11.5)	
Sapien 3	43/356 (12.1)	4/43 (9.3)	39/313 (12.5)	
Label size of new valve, mm	23 (23; 26)	29 (26; 29)	23 (23; 26)	< 0.001
Need for 2nd THV, *n* (%)	4/356 (1.1)	0/43 (0)	4/313 (1.3)	1.000
Predilatation, *n* (%)	232/338	29/38 (76.3)	203/300 (67.7)	0.279
Postdilatation, *n* (%)	63/338 (18.6)	7/38 (18.4)	56/300 (18.7)	0.971
Bioprosthetic valve fracture, *n* (%)	0/313 (0)	n.a	0/313 (0)	n.a
Coronary Protection				
BASILICA, *n* (%)	0	0	0	
Chimney stenting, *n* (%)	4/356 (1.1)	1/43 (2.3)	3/313 (1.0)	0.404
Coronary obstruction, *n* (%)	1/356 (0.3)	1/43 (2.3)	0/313 (0)	n.a
Echo before discharge				
Mean gradient [mmHg]	15 (10; 21) *n* = 349	10 (6; 16) *n* = 41	15 (11; 22) *n* = 308	< 0.001
Mean gradient ≥ 20 mmHg, *n* (%)	107/349 (30.7)	4/41 (9.8)	103/308 (33.4)	0.002
Moderate aortic regurgitation after ViV*, *n* (%)	11/349 (3.2)	3/40 (7.5)	8/309 (2.6)	0.120

Values are *n* (%) or median (IQR). ViV indicates valve-in-valve; *THV* transcatheter heart valve; *BASILICA* Bioprosthetic or Native Aortic Scallop Intentional Laceration to Prevent Iatrogenic Coronary Artery Obstruction; *n.a.* not applicable

^*^no severe AR occurred

### Primary outcome measures and their predictors

The rates of technical success (83.7% versus 79.9%), device success (67.4% versus 54.3%), and early safety (74.4% versus 66.5%) were not significantly different between SL and ST (Table 3). The 30-day mortality rate was 7.0% in SL and 2.6% in ST (*p* = 0.136). A detailed description of the causes of death until day 30 is provided in Online Table II. In a binary logistic regression analysis, the odds ratios were OR 0.58, 95%CI 0.21; 1.57 for technical success, OR 1.32, 95%CI 0.60; 2.94 for device success, and OR 1.67, 95%CI 0.78; 3.58 for early safety comparing SL versus ST (Online Tables III–V). Predictors of technical success were the use of a self-expanding versus balloon-expandable THV (OR 0.42, 95%CI 0.20; 0.90), baseline LV-EF (OR 1.03, 95%CI 1.00; 1.06), and treatment period 2016–2022 versus 2007–2015 (OR 2.19, 95%CI 1.23; 3.89) (Online Table III). Factors associated with device success included previous stroke (OR 0.49, 95%CI 0.25; 0.95), an immunocompromised status (OR 0.38, 95%CI 0.14; 0.99), TID < 20 mm (OR 0.54, 95%CI 0.35; 0.85), and treatment period 2016–2022 versus 2007–2015 (OR 1.74, 95%CI 1.11; 2.71), whereas early safety was associated with previous stroke (OR 0.46, 95%CI 0.24; 0.90), an immunocompromised status (OR 0.30, 95%CI 0.12; 0.76), and use of a self-expanding valve (OR 0.44, 95%CI 0.24; 0.82) (Online Tables IV and V).

**Table 3:** VARC-3-defined endpoints and complications after ViV-TAVI according to valve type

	Total ViV cohort (*n* = 356)	Stentless valve (*n* = 43)	Stented valve (*n* = 313)	*p*-value
VARC-3-defined composite endpoints				
30-day mortality, *n* (%)	11/356 (3.1)	3/43 (7.0)	8/313 (2.6)	0.136
Technical success (VARC-3), *n* (%)	286/356 (80.3)	36/43 (83.7)	250/313 (79.9)	0.552
Device success (VARC-3), *n* (%)	198/354 (55.9)	29/43 (67.4)	169/311 (54.3)	0.105
Early safety (VARC-3), *n* (%)	240/356 (67.4)	32/43 (74.4)	208/313 (66.5)	0.296
VARC-3- defined complications				
VARC-3 myocardial infarction, *n* (%)	8/356 (2.2)	2/43 (4.7)	6/313 (1.9)	0.250
VARC-3 Stroke, *n* (%)	14/356 (3.9)	0/43 (0)	14/313 (4.5)	0.391
Major, *n* (%)	11/356 (3.1)	0/43 (0)	11/313 (3.5)	0.373
Minor, *n* (%)	3/356 (0.8)	0/43 (0)	3/313 (1.0)	1.000
VARC-3 Bleeding, *n* (%)	60/356 (16.9)	6/43 (14.0)	54/313 (17.3)	0.588
Type 1, *n* (%)	21/356 (5.9)	3/43 (7.0)	18/313 (5.8)	0.729
Type 2, *n* (%)	20/356 (5.6)	0/43 (0)	20/313 (6.4)	0.149
Type 3, *n* (%)	17/356 (4.8)	3/43 (7.0)	14/313 (4.5)	0.444
Type 4, *n* (%)	2/356 (0.6)	0/43 (0)	2/313 (0.6)	1.000
VARC-3 Kidney, *n* (%)	28/356 (7.9)	5/43 (11.6)	23/313 (7.3)	0.360
Stage 1, *n* (%)	15/356 (4.2)	3/43 (7.0)	12/313 (3.8)	0.406
Stage 2, *n* (%)	5/356 (1.4)	0/43 (0)	5/313 (1.6)	1.000
Stage 3, *n* (%)	3/356 (0.8)	1/43 (2.3)	2/313 (0.6)	0.321
Stage 4, *n* (%)	5/356 (1.4)	1/43 (2.3)	4/313 (1.3%)	0.477
VARC-3 Access site, *n* (%)	63/356 (17.7)	5/43 (11.6)	58/313 (18.5)	0.266
Major, *n* (%)	21/356 (5.9)	1/43 (2.3)	20/313 (6.4)	0.490
Minor, *n* (%)	42/356 (11.8)	4/43 (9.3)	38/313 (12.1)	0.801
Need for new pacemaker, *n* (%)	26/288 (9.0)	2/34 (5.9)	24/254 (9.4)	0.751

Values are *n* (%). VARC indicates Valve Academic Research Consortium

### Secondary outcomes

Secondary outcomes are displayed in Table 3 showing comparable results between groups with regard to VARC-3 defined myocardial infarction, stroke, bleeding, acute kidney injury, access site complication, and need for new permanent pacemaker implantation. There was no conversion to cardiac surgery.

### Long-term all-cause mortality and its predictors

The median duration of follow-up was 599 days (IQR 276–1210) with no significant differences between SL (546 days, IQR 175; 1370) and ST (609 days, IQR 337; 1156) (*p* = 0.557).

**Fig. 1:**
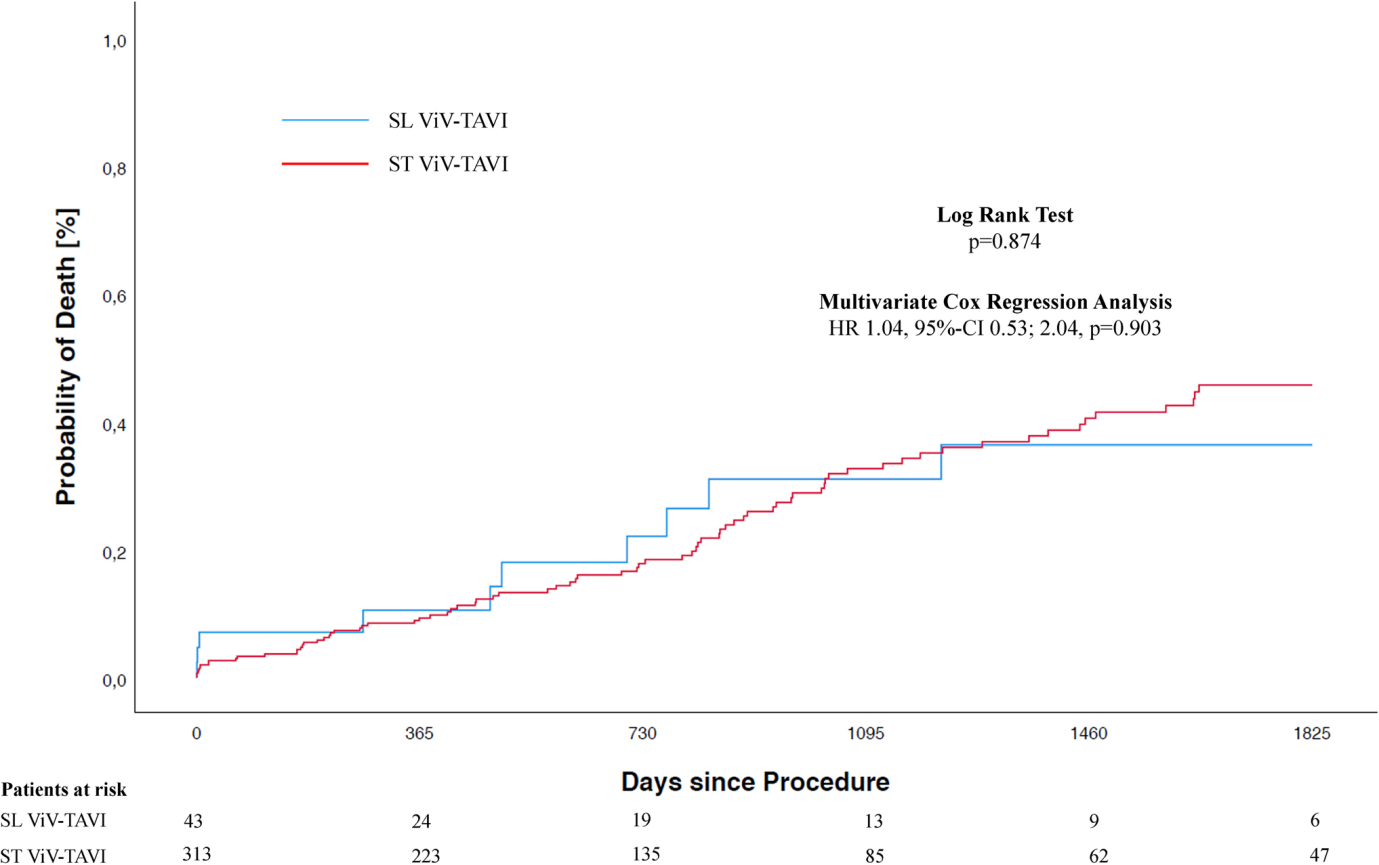
Kaplan–Meier estimates of mortality in patients undergoing valve-in-valve transcatheter aortic valve implantation (ViV-TAVI) into stentless (SL) compared with stented (ST) failed aortic bioprostheses

All-cause mortality at 1-year, 3-year, and 5-year follow-up was 9.3%, 20.9%, and 23.3% for SL and 8.0%, 20.1%, and 24.6% for ST, respectively (according *p*-values by log-rank test 0.627, 0.841, 0.874) (Fig. 1).

In a multivariate Cox regression analysis, independent predictors for 5-year all-cause mortality were STS-PROM (HR per 1% increase 1.04, 95%CI 1.02; 1.06), a stenotic mode of bioprosthetic failure (HR 1.82, 95%CI 1.16; 2.87), and a VARC-3-defined MI (HR 5.27, 95%CI 2.07; 13.43). ViV-TAVI for SL versus ST was not significantly associated with 5-year all-cause mortality (HR 1.04, 95%CI 0.53; 2.04) (Online Table VI).

### Symptomatic improvement

**Fig. 2:**
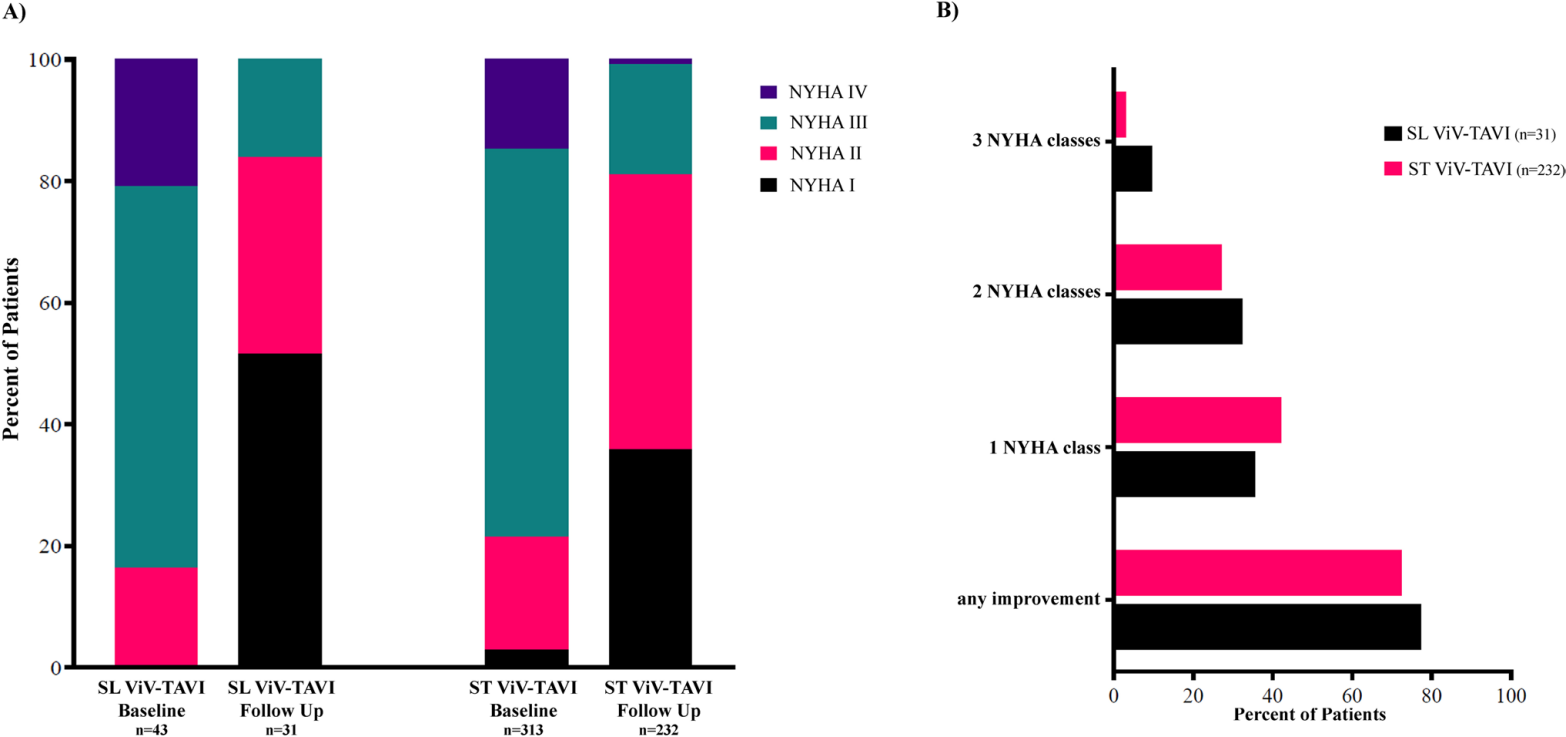
NYHA class at baseline (*p*-value for SL vs ST = 0.513) and after 1 year of follow-up (*p*-value for SL vs ST = 0.361) (**A**). Percentage of patients showing any improvement of NYHA class (*p*-value for SL vs ST = 0.835) and categorized by an improvement in 1, 2, or 3 NYHA classes (all *p*-values for SL vs ST > 0.05) (**B**)

At baseline, most of the patients were severely symptomatic with 79.2% of the whole cohort suffering dyspnea according to NYHA III/IV with no significant differences between groups (Table 1 and Fig. 2). After 1 year, NYHA classification was available in 263 patients. There was substantial improvement in both SL and ST without significant difference between groups (Fig. 2A). The proportion of patients with an improvement of at least one NYHA class was comparable between SL (23/31, 74.2%) and ST (168/232, 72.4%) (*p* = 0.835) (Fig. 2B).

### Sensitivity analysis

To account for changes over time, a sensitivity analysis according to two treatment periods (2007–2015 and 2016–2022) was performed. The results are shown in Online Tables VII–IX and Online Fig. 1. These analyses revealed that the risk profile decreased over the years, whereas patient´s age was comparable. VARC-3 defined endpoints partially improved over the years with higher rates of technical/device success and early safety as well as reduced peri-interventional complications and lower 30-day mortality in the more recent time. However, there were no statistically significant differences in the outcome between SL and ST in either period.

## Discussion

We aimed to compare characteristics and outcomes of patients with stentless versus stented aortic bioprosthetic device failure undergoing ViV-TAVI in a cohort of patients receiving a significant portion of contemporary TAVI devices applying the updated VARC-3 outcome definitions. The main findings of our multicenter observational analysis are as follows: (1) SL ViV-TAVI contributed to roughly 12% of all ViV-TAVI procedures. (2) The mode of failure differed between SL and ST with a higher proportion of aortic regurgitation in SL and a higher rate of aortic stenosis in ST. (3) The VARC-3 defined outcomes technical success, device success, and early safety were not significantly different between SL and ST. (4) The mortality over the course of 5 years did not differ between SL and ST and was predicted by the baseline risk of the patient, a stenotic mode of aortic bioprosthetic device failure, and periprocedural myocardial infarction.

In SAVR, stentless aortic bioprostheses are used in roughly 10% of all implanted bioprostheses. [4] Since failure rates are comparable between SL and ST bioprostheses [4], the rate of roughly 12% SL in our cohort is in the expected range. Moreover, in the so far largest series comparing SL with ST ViV-TAVI, SL contributed to 18% of all cases. This slightly higher number might be in particular related to the selection bias within those registries and regional differences in utilization of surgical valve type selection for the primary operation.

The mode of bioprosthetic valve failure differed significantly between SL and ST with a higher proportion of aortic regurgitation in SL. Despite that, the rate of a stenotic mode of failure was the main mechanism in both SL and ST (39.5% versus 56.5%). This high rate of restenosis in SL is in contrast to other published series with rates reported to be about 18%. One reason might be the high proportion of the *Sorin Freedom* in our cohort (62.8%) which is known to develop a stenotic failure due to severe calcification of the leaflets [14, 15]. This also supports the abovementioned aspect that a different regional surgical valve type selection might affect the results of ViV-TAVI registries.

To the best of our knowledge, this is the first analysis comparing SL versus ST ViV-TAVI applying systematically VARC-3 defined composite endpoints. The composite endpoint technical success was introduced in the latest VARC-3 definitions to capture the immediate success of a procedure, which is measured at the time of leaving the procedure room and encompasses the true technical safety of the device and its delivery. [11] This endpoint was reached in 80.3% in our cohort with no significant differences between SL and ST. The main reasons for not achieving technical access were related to major access site and cardiac structural complications leading to other interventions or operations. Since this endpoint has just been proposed in the latest VARC-3 publication, no comparisons to other cohorts can be made at this time point.

Device success, which now includes technical success according to VARC-3, was achieved in 55.9% of the whole cohort with numerically higher but not statistically significant values in SL compared with ST. Not to achieve device success was mainly related to significantly higher residual gradients after ST ViV-TAVI with a higher proportion of patients having residual gradients ≥ 20 mmHg. The proportion of patients having residual gradients ≥ 20 mmHg in our cohort was roughly 30%, which is comparable to the rates reported in the current literature [16, 17]. However, higher gradients did not affect 5-year mortality, a finding which is also consistent with the current literature showing no impact on mortality up to 10 years of follow-up after ViV-TAVI [7, 18]. Bioprosthetic valve fracture, known to reduce residual gradients in ViV-TAVI [19], was not used in our cohort and might be a missed chance in selected cases; however, the high proportion of ST bioprostheses in our cohort that cannot be fractured (e.g., the Hancock family) might limit the overall effect of such a procedure.

Early safety was reached in 67.4% of all ViV-TAVI, again with no significant differences between SL and ST. The main reasons for not achieving this composite endpoint were due to bleeding and access site complications. With regard to the individual VARC-3 defined endpoints, no significant differences were found between groups. In the so far largest analysis comparing SL and ST ViV-TAVI [10], also no significant differences were found for access site or bleeding complications, stroke rates, or acute kidney injury. However, higher rates of procedural complications, e.g., coronary obstruction, need for a 2nd THV, and PVL, were observed for SL compared with ST ViV-TAVI [10]. This was not seen in our analysis. Indeed, coronary obstruction occurred just once (0.3%) and a 2nd THV was needed in only 4 cases (1.1%) in our analysis, whereas Duncan et al. reported corresponding rates of 2.3% and 4.2% with a predominance in SL compared to ST ViV-TAVI [10]. Moreover, in the report of Duncan et al. the need for a second THV was overall higher and pronounced in SL ViV-TAVI, which was not the case in our analysis. The reasons for those differences are likely multifactorial including the treatment of a more contemporary cohort which was better characterized by improved preoperative imaging and the use of the latest generation of TAVI prostheses. Moreover, a learning curve of the individual operators and the interventional teams is an important factor contributing to improved procedural outcomes in TAVI. [20, 21] This is supported by the fact that treatment period (2016–2022 versus 2007–2015) was an independent predictor for technical and device success in our analysis. Additionally, our sensitivity analysis provides evidence that patients treated more recently had better VARC-3 defined outcomes with no differences between SL and ST, indicating that it was not the initial type of bioprosthesis affecting the outcome. Improved outcomes and lower complication rates over time have been described in other TAVI cohorts. [22, 23] Interestingly, the use of a self-expanding THV was associated with a lower technical success and early safety which might be related to higher rates of valve embolization, new permanent pacemaker implantation, and access site complications with self-expanding versus balloon-expandable THVs (data not shown).

Mortality after ViV-TAVI has been reported in several cohorts up to three years. A small multicenter assessment of 116 ViV-TAVI patients (mean age of 76.0 ± 11.0 years, mean STS score 8.0 ± 5.1%) reported a 5-year mortality rate of 32.1%. [24] In a cohort of 356 high-risk patients from the PARTNER trial (mean age of 78.9 ± 10.2 years, mean STS score of 9.1 ± 4.7%), the estimated 3-year mortality rate was 32.7%. [25] The report from the CoreValve US Expanded Use Study with a total of 226 extreme-risk patients (mean age of 76.7 ± 10.8 years, mean STS score of 9.0 ± 6.7%) was also recently published and reported a 3-year mortality rate of 27.7%. [26] The data from the VIVID Registry including 1006 high-risk patients (mean age of 77.7 ± 9.7 years, median STS score 7.3% (IQR 4.2; 12.0)) revealed a 3-year mortality rate of 27.0% and an 8-year mortality rate of 62% after ViV-TAVI. [7] In our cohort, mean age was 78.3 ± 6.8 years, mean STS score was 9.5 ± 7.7% (median 7.0% (IQR 4.6; 11.4), and mortality rate at 1, 3, and 5 years was 8.1%, 20.2%, and 24.4%, respectively. Despite a similar age and risk profile, the mortality was lower in our cohort which again might be attributable to the reasons mentioned above including appropriate patient selection, improved imaging, use of latest devices, and greater operator experience in a more contemporary patient cohort. With regard to the comparison of SL versus ST ViV-TAVI, no significant mortality difference was detected between groups. This is in line with the results published by Duncan et al. who also found no mortality difference between SL and ST ViV-TAVI after 1 year despite higher periprocedural complications in SL ViV-TAVI. [10]

Predictors of 5-year all-cause mortality were related to the periprocedural risk assessed by the STS-PROM, a stenotic mode of bioprosthetic valve failure and periprocedural myocardial infarction in our analysis. The type of the initial surgical valve (SL versus ST) was not associated with 5-year outcome. This is in line with former studies showing no influence of the initial surgical valve on outcomes in multivariate analysis. [7, 10] The increased mortality risk in patients presenting with restenosis has also been described in former reports. [27] Interestingly, we found no increased mortality risk in patients with small aortic bioprostheses which is in contrast to the long-term analysis from the VIVID registry. [7] Valve size was not included in the multivariable analysis of the CoreValve US Expanded Use Study [26] and patients with a label size < 21 mm were excluded in the PARTNER study. [25] Therefore, further studies are necessary to evaluate the impact of initial valve size on the outcome of patients undergoing ViV-TAVI, in particular with a focus on long-term outcome.

Symptomatic improvement at 1 year according to NYHA class occurred in roughly 75% of the patients with available data in our analysis regardless of the primary surgical valve type. This is comparable to other reports showing that about 70 to 90% of ViV-TAVI patients are in NYHA class I/II after one year. [8, 27, 28].

### Limitations

We are well aware of certain limitations in our study. Although data were analyzed from a prospective registry including real-world, consecutive patients, all biases inherent to a retrospectively evaluated, unmonitored multicenter registry have to be taken into account while interpreting these data. The time period lasted from 2007 to 2022, a time in which TAVI experienced many technical and procedural changes. Despite including operation period into the statistical models, influences by latest device iteriations, improved imaging, and patient selection as well as operator experience are of importance while interpreting these results. Moreover, this registry recorded only ViV-TAVI cases but not the rate of redo surgery for degenerated aortic bioprostheses during the same time period; therefore, no information on treatment selection and potential temporal changes can be provided. Unfortunately, clinical and echocardiographic follow-up is limited in our registry and no meaningful conclusions can be made on structural valve deterioration and bioprosthetic valve failure.

## Conclusion

SL and ST ViV-TAVI led to comparable short-term outcomes according to VARC-3- defined endpoints and similar mortality rates up to 5 years of follow-up. Against the background of increased gradients, in particular in ST ViV-TAVI, long-term studies are of importance to determine the clinical relevance with regard to repeated structural valve deterioration and bioprosthetic valve failure.

## Supplementary Information

Below is the link to the electronic supplementary material.

Supplementary file1 (DOCX 203 KB)

## Data Availability

The data underlying this paper will be shared upon reasonable request to the corresponding author and lead authors of each participating center.

## Abbreviations

HR: Hazard ratio
IQR: Interquartile range
OR: Odds ratio
SL: Stentless aortic bioprosthesis
ST: Stented aortic bioprosthesis
STS-PROM: Society of Thoracic Surgeons Predicted Risk of Mortality
THV: Transcatheter heart valve
VARC: Valve Academic Research Consortium
ViV-TAVI: Valve-in-valve transcatheter aortic valve implantation
