# Supplementary material for: VARC-3 defined outcome of valve-in-valve transcatheter aortic valve implantation in stentless compared with stented aortic bioprostheses
Source: Clin Res Cardiol. 2023 Dec 11;114(3):291–301. doi: 10.1007/s00392-023-02347-5 (PMC11913896; doi:10.1007/s00392-023-02347-5)

**Supplemental Materials**

**VARC-3 defined outcome of valve-in-valve transcatheter aortic valve implantation in stentless compared with stented aortic bioprostheses**

**Online Table I.** Failed valve models by structural type

**Online Table II.** Causes of death until day 30

**Online Table III.** Binary logistic regression for Technical Success, Univariate and multivariate predictors of technical success derived from baseline characteristics

**Online Table IV.** Binary logistic regression Device Success, Univariate and multivariate predictors of technical success derived from baseline characteristics

**Online Table V.** Binary logistic regression Early Safety, Univariate and multivariate predictors of technical success derived from baseline characteristics

**Online Table VI.** Univariate and multivariate predictors of 5-year mortality derived from baseline and procedural characteristics

**Online Table VII:** Baseline characteristics according to valve type and treatment period

**Online Table VIII.** Procedural characteristics and in-hospital hemodynamic outcomes according to valve type and treatment period

**Online Table IX.** VARC-3-defined endpoints and complications after ViV-TAVI according to valve type and treatment period

**Online Figure 1.** Kaplan-Meier estimates of mortality in patients undergoing valve-in-valve transcatheter aortic valve implantation (ViV-TAVI) into stentless (SL) compared with stented (ST) failed aortic bioprostheses according to treatment period.

**Online Table I.** Failed valve models by structural type

| **Stentless (n = 43)** | **Frequency** | **%** |  | **Stented (n = 313)** | **Frequency** | **%** |
| --- | --- | --- | --- | --- | --- | --- |
| Sorin Freedom | 27 | 62.8 |  | Medtronic Hancock II | 61 | 19.5 |
| Shelhigh | 7 | 16.3 |  | Sorin Mitroflow | 56 | 17.9 |
| St. Jude Toronto | 4 | 9.3 |  | Carpentier Edwards Perimount | 50 | 16.0 |
| Freestyle Aortic Root | 1 | 2.3 |  | Carpentier Edwards Perimount Magna | 43 | 13.7 |
| Vascutek Elan | 1 | 2.3 |  | St. Jude Epic | 37 | 11.8 |
| Cryolife Homograft | 1 | 2.3 |  | Unknown | 14 | 4.4 |
| Baxter-Edwards | 1 | 2.3 |  | Medtronic Hancock | 11 | 3.5 |
| Unknown | 1 | 2.3 |  | Medtronic Mosaic | 11 | 3.5 |
|  |  |  |  | Sorin Soprano | 10 | 3.2 |
|  |  |  |  | Baxter-Edwards | 6 | 1.9 |
|  |  |  |  | St. Jude Trifecta | 5 | 1.6 |
|  |  |  |  | Sorin Perceval | 4 | 1.3 |
|  |  |  |  | Carpentier Edwards Perimount Magna Ease | 3 | 1.0 |
|  |  |  |  | St. Jude Biocor | 2 | 0.6 |

**Online Table II.** Causes of death until day 30

| **Causes of Death** | **Stentless valve** | **Stented valve** | |  |
| --- | --- | --- | --- | --- |
| Ventricle perforation | 1 | | 0 | |
| Retroperitoneal bleeding | 0 | | 1 | |
| Other access related bleedings | 0 | | 2 | |
| Stroke | 0 | | 2 | |
| Myocardial infarction | 2 | | 0 | |
| Sepsis | 0 | | 2 | |
| Undetermined | 0 | | 1 | |

**Online Table III.** Binary logistic regression for Technical Success, Univariate and multivariate predictors of technical success derived from baseline characteristics

|  | **Univariate** | | **Multivariate** | |
| --- | --- | --- | --- | --- |
|  | **HR (95%-CI)** | **p-value** | **HR (95%-CI)** | **p-value** |
| **Baseline factors** |  |  |  |  |
| Age [per 1 year increase] | **0.98 (0.94; 1.02)** | **0.314** | 0.99 (0.95; 1.04) | 0.658 |
| Male sex | **1.62 (0.96; 2.74)** | **0.071** | 1.05 (0.59; 1.90) | 0.861 |
| Body mass index [per 1 kg/m² increase] | 1.00 (0.95; 1.05) | 0.969 |  |  |
| STS-PROM [per 1% increase] | 0.99 (0.96; 1.02) | 0.612 |  |  |
| NYHA III/IV | 1.17 (0.62; 2.18) | 0.634 |  |  |
| Coronary artery disease | 1.44 (0.86; 2.44) | 0.170 |  |  |
| Previous myocardial infarction | 0.63 (0.30; 1.33) | 0.223 |  |  |
| Previous PCI | 1.02 (0.53; 1.95) | 0.958 |  |  |
| Previous CABG | 1.21 (0.69; 2.12) | 0.505 |  |  |
| Atrial fibrillation | 0.78 (0.46; 1.31) | 0.344 |  |  |
| Diabetes mellitus | **1.86 (1.04; 3.34)** | **0.038** | 1.13 (0.64; 2.03) | 0.670 |
| Previous stroke | 0.63 (0.31; 1.30) | 0.209 |  |  |
| PAD | 0.91 (0.46; 1.78) | 0.776 |  |  |
| Carotid stenosis | 0.89 (0.45; 1.75) | 0.731 |  |  |
| COPD | 1.01 (0.58; 1.76) | 0.969 |  |  |
| CKD >3b | 1.13 (0.65; 1.97) | 0.659 |  |  |
| Immunocompromised status | 0.59 (0.22; 1.58) | 0.295 |  |  |
| Pacemaker prior to ViV | 1.04 (0.53; 2.04) | 0.900 |  |  |
| Left Ventricular Ejection Fraction [per 1% decrease] | **1.04 (1.02; 1.07)** | **0.002** | **1.03 (1.00; 1.06)** | **0.025** |
| Stentless vs. stented prosthesis | **1.30 (0.55; 3.05)** | **0.552** | 0.58 (0.21; 1.57) | 0.279 |
| Mode of Failure. Stenosis | 1.25 (0.74; 2.11) | 0.400 |  |  |
| TID <20 mm | **0.44 (0.26; 0.77)** | **0.004** | 0.58 (0.33; 1.04) | 0.068 |
| Moderate/severe PPM | 1.03 (0.33; 3.20) | 0.958 |  |  |
| Self-expanding vs balloon-expandable THV | **0.51 (0.25; 1.04)** | **0.063** | **0.42 (0.20; 0.90)** | **0.025** |
| Treatment period 2016-2022 vs 2007-2015 | **2.02 (1.19; 3.45)** | **0.010** | **2.19 (1.23; 3.89)** | **0.008** |

CI indicates confidence interval; ViV, valve-in-valve; STS-PROM, Society of Thoracic Surgeons Predicted Risk of Mortality; NYHA, New York Heart Association; PCI, percutaneous coronary intervention; CABG, coronary artery bypass graft; PAD, peripheral artery disease; COPD, chronic obstructive pulmonary disease; CKD, chronic kidney disease; TID, true internal diameter; PPM, patient prosthesis mismatch; THV, transcatheter heart valve

**Online Table IV.** Binary logistic regression Device Success, Univariate and multivariate predictors of technical success derived from baseline characteristics

|  | **Univariate** | | **Multivariate** | |
| --- | --- | --- | --- | --- |
|  | **HR (95%-CI)** | **p-value** | **HR (95%-CI)** | **p-value** |
| **Baseline factors** |  |  |  |  |
| Age [per 1 year increase] | **1.00 (0.97; 1.04)** | **0.824** | 1.01 (0.97; 1.04) | 0.767 |
| Male sex | **1.17 (0.77; 1.79)** | **0.469** | 0.88 (0.54; 1.42) | 0.594 |
| Body mass index [per 1 kg/m² increase] | 0.97 (0.93; 1.01) | 0.111 |  |  |
| STS-PROM [per 1% increase] | 1.01 (0.98; 1.04) | 0.529 |  |  |
| NYHA III/IV | 0.99 (0.59; 1.66) | 0.964 |  |  |
| Coronary artery disease | 0.84 (0.55; 1.28) | 0.419 |  |  |
| Previous myocardial infarction | 0.81 (0.42; 1.55) | 0.519 |  |  |
| Previous PCI | 0.85 (0.51; 1.43) | 0.546 |  |  |
| Previous CABG | 1.24 (0.80; 1.93) | 0.345 |  |  |
| Atrial fibrillation | 0.90 (0.58; 1.38) | 0.614 |  |  |
| Diabetes mellitus | 0.92 (0.60; 1.42) | 0.704 |  |  |
| Previous stroke | **0.59 (0.31; 1.11)** | **0.099** | **0.49 (0.25; 0.95)** | **0.033** |
| PAD | 0.91 (0.46; 1.78) | 0.776 |  |  |
| Carotid stenosis | 0.84 (0.49; 1.47) | 0.548 |  |  |
| COPD | 0.84 (0.54; 1.30) | 0.428 |  |  |
| CKD >3b | 1.29 (0.83; 2.01) | 0.255 |  |  |
| Immunocompromised status | **0.37 (0.15; 0.95)** | **0.038** | **0.38 (0.14; 0.99)** | **0.047** |
| Pacemaker prior to ViV | 1.16 (0.67; 1.98) | 0.503 |  |  |
| LVEF [per 10% decrease] | **1.02 (1.00; 1.04)** | **0.015** | 1.02 (1.00; 1.04) | 0.104 |
| Stentless vs. stented prosthesis | **1.74 (0.89; 3.42)** | **0.108** | 1.32 (0.60; 2.94) | 0.489 |
| Mode of Failure. Stenosis | 1.30 (0.85; 1.98) | 0.228 |  |  |
| TID <20 mm | **0.56 (0.36; 0.86)** | **0.008** | **0.54 (0.35; 0.85)** | **0.007** |
| Moderate/severe PPM | 1.24 (0.49; 3.13) | 0.653 |  |  |
| Self-expanding vs balloon-expandable THV | 1.08 (0.65; 1.79) | 0.760 |  |  |
| Treatment period 2016-2022 vs 2007-2015 | **1.50 (0.96; 2.35)** | **0.073** | **1.74 (1.11; 2.71)** | **0.015** |

CI indicates confidence interval; ViV, valve-in-valve; STS-PROM, Society of Thoracic Surgeons Predicted Risk of Mortality; NYHA, New York Heart Association; PCI, percutaneous coronary intervention; CABG, coronary artery bypass graft; PAD, peripheral artery disease; COPD, chronic obstructive pulmonary disease; CKD, chronic kidney disease; TID, true internal diameter; PPM, patient prosthesis mismatch; THV, transcatheter heart valve

**Online Table V.** Binary logistic regression Early Safety, Univariate and multivariate predictors of technical success derived from baseline characteristics

|  | **Univariate** | | **Multivariate** | |
| --- | --- | --- | --- | --- |
|  | **HR (95%-CI)** | **p-value** | **HR (95%-CI)** | **p-value** |
| **Baseline factors** |  |  |  |  |
| Age [per 1 year increase] | **1.01 (0.98; 1.04)** | **0.668** | 1.01 (0.98; 1.05) | 0.439 |
| Male sex | **0.97 (0.62; 1.52)** | **0.900** | 0.89 (0.55; 1.45) | 0.634 |
| Body mass index [per 1 kg/m² increase] | 0.99 (0.94; 1.03) | 0.532 |  |  |
| STS-PROM [per 1% increase] | 0.99 (0.96; 1.02) | 0.393 |  |  |
| NYHA III/IV | 0.85 (0.49; 1.48) | 0.556 |  |  |
| Coronary artery disease | 1.10 (0.70; 1.72) | 0.675 |  |  |
| Previous myocardial infarction | 0.65 (0.33; 1.26) | 0.200 |  |  |
| Previous PCI | 0.96 (0.55; 1.66) | 0.879 |  |  |
| Previous CABG | 1.21 (0.76; 1.94) | 0.419 |  |  |
| Atrial fibrillation | 0.79 (0.51; 1.24) | 0.311 |  |  |
| Diabetes mellitus | **1.52 (0.94; 2.43)** | **0.085** | 1.56 (0.95; 2.56) | 0.076 |
| Previous stroke | **0.56 (0.30; 1.05)** | **0.072** | **0.46 (0.24; 0.90)** | **0.023** |
| PAD | **0.61 (0.35; 1.07)** | **0.086** | 0.61 (0.34; 1.10) | 0.098 |
| Carotid stenosis | 0.83 (0.47; 1.48) | 0.535 |  |  |
| COPD | 1.22 (0.75; 1.96) | 0.422 |  |  |
| CKD >3b | 1.24 (0.77; 1.98) | 0.377 |  |  |
| Immunocompromised status | **0.34 (0.14; 0.83)** | **0.017** | **0.30 (0.12; 0.76)** | **0.011** |
| Pacemaker prior to ViV | 1.10 (0.62; 1.95) | 0.739 |  |  |
| LVEF [per 1% decrease] | **1.02 (1.00; 1.04)** | **0.038** | 1.02 (1.00; 1.04) | 0.069 |
| Stentless vs. stented prosthesis | **1.47 (0.71; 3.03)** | **0.298** | 1.67 (0.78; 3.58) | 0.183 |
| Mode of Failure. Stenosis | 0.87 (0.55; 1.35) | 0.527 |  |  |
| TID <20 mm | 0.78 (0.49; 1.22) | 0.268 |  |  |
| Moderate/severe PPM | 0.94 (0.36; 2.41) | 0.890 |  |  |
| Self-expanding vs balloon-expandable THV | **0.47 (0.26; 0.85)** | **0.012** | **0.44 (0.24; 0.82)** | **0.009** |
| Treatment period 2016-2022 vs 2007-2015 | 1.15 (0.75; 1.78) | 0.522 |  |  |

CI indicates confidence interval; ViV, valve-in-valve; STS-PROM, Society of Thoracic Surgeons Predicted Risk of Mortality; NYHA, New York Heart Association; PCI, percutaneous coronary intervention; CABG, coronary artery bypass graft; PAD, peripheral artery disease; COPD, chronic obstructive pulmonary disease; CKD, chronic kidney disease; TID, true internal diameter; PPM, patient prosthesis mismatch; THV, transcatheter heart valve

**Online Table VI.** Univariate and multivariate predictors of 5-year mortality derived from baseline and procedural characteristics

|  | **Univariate** | | **Multivariate** | |
| --- | --- | --- | --- | --- |
|  | **HR (95%-CI)** | **p-value** | **HR (95%-CI)** | **p-value** |
| **Baseline factors** |  |  |  |  |
| Age [per 1 year increase] | **1.01 (0.97; 1.04)** | **0.786** | 1.01 (0.97; 1.04) | 0.696 |
| Male sex | **0.83 (0.54; 1.26)** | **0.377** | 0.97 (0.60; 1.57) | 0.973 |
| Body mass index [per 1 kg/m² increase] | 1.02 (0.98; 1.07) | 0.351 |  |  |
| STS-PROM [per 1% increase] | **1.04 (1.02; 1.06)** | **<0.001** | **1.04 (1.02; 1.06)** | **<0.001** |
| NYHA III/IV | 1.12 (0.66; 1.90) | 0.676 |  |  |
| Coronary artery disease | 1.33 (0.86; 2.05) | 0.200 |  |  |
| Previous myocardial infarction | 1.41 (0.75; 2.66) | 0.286 |  |  |
| Previous PCI | 0.75 (0.42; 1.32) | 0.316 |  |  |
| Previous CABG | 1.04 (0.67; 1.62) | 0.855 |  |  |
| Atrial fibrillation | 1.27 (0.83; 1.95) | 0.273 |  |  |
| Diabetes mellitus | 1.12 (0.73; 1.72) | 0.606 |  |  |
| Previous stroke | 0.90 (0.47; 1.75) | 0.763 |  |  |
| PAD | 1.34 (0.79; 2.24) | 0.276 |  |  |
| Carotid stenosis | 0.73 (0.39; 1.38) | 0.332 |  |  |
| COPD | 0.93 (0.59; 1.45) | 0.743 |  |  |
| CKD >3b | 0.98 (0.63; 1.52) | 0.927 |  |  |
| Immuncompromised status | **2.29 (1.05; 4.99)** | **0.037** | 1.18 (0.42; 3.32) | 0.756 |
| Pacemaker prior to ViV | 1.43 (0.88; 2.32) | 0.147 |  |  |
| LVEF [%] | 1.01 (1.00; 1.03) | 0.129 |  |  |
| Treatment period 2016-2022 vs 2007-2015 | 1.15 (0.75; 1.78) | 0.522 |  |  |
| Stentless vs. stented prosthesis | **0.95 (0.49; 1.83)** | **0.874** | 1.04 (0.53; 2.04) | 0.903 |
| Mode of Failure. Stenosis | **2.01 (1.29; 3.14)** | **0.002** | **1.82 (1.16; 2.87)** | **0.009** |
| Moderate/severe PPM | 1.75 (0.90; 3.42) | 0.102 |  |  |
| TID <20 mm | 1.16 (0.75; 1.78) | 0.511 |  |  |
| **Procedural factors** |  |  |  |  |
| Self-expanding vs balloon-expandable THV | 1.28 (0.72; 2.27) | 0.403 |  |  |
| Post-procedural gradient ≥20mmHg | 1.04 (0.65; 1.66) | 0.878 |  |  |
| Moderate paravalvular aortic regurgitation | 1.55 (0.57; 4.23) | 0.396 |  |  |
| VARC-MI | **6.64 (2.64; 16.71)** | **<0.001** | **5.27 (2.07; 13.43)** | **0.001** |
| VARC Stroke | **2.25 (0.98; 5.16)** | **0.057** | 1.53 (0.61; 3.86) | 0.364 |
| VARC Bleeding | 1.33 (0.78; 2.26) | 0.292 |  |  |
| VARC Access site complication | 1.17 (0.68; 2.01) | 0.578 |  |  |
| VARC Kidney injury | **1.72 (0.91; 3.24)** | **0.092** | 1.06 (0.53; 2.11) | 0.870 |
| New PM/ICD | 1.35 (0.54; 3.38) | 0.523 |  |  |

CI indicates confidence interval; ViV, valve-in-valve; STS-PROM, Society of Thoracic Surgeons Predicted Risk of Mortality; NYHA, New York Heart Association; PCI, percutaneous coronary intervention; CABG, coronary artery bypass graft; PAD, peripheral artery disease; COPD, chronic obstructive pulmonary disease; CKD, chronic kidney disease; TID, true internal diameter; PPM, patient prosthesis mismatch; THV, transcatheter heart valve; VARC, Valve Academic Research Consortium; MI, myocardial infarction; PM, pacemaker; ICD, implantable cardioverter defibrillator

**Online Table VII:** Baseline characteristics according to valve type and treatment period

|  | **2007 – 2015** | | | **2016-2022** | | |
| --- | --- | --- | --- | --- | --- | --- |
|  | **Stentless valve**  **(n = 14)** | **Stented valve**  **(n = 155)** | **p-value** | **Stentless valve**  **(n = 29)** | **Stented valve**  **(n = 158)** | **p-value** |
| Age [years] | 75 (73; 79) | 79 (74; 82) | 0.059 | 79 (75, 82) | 80 (76; 83) | 0.303 |
| Male sex, n (%) | 11 (78.6) | 83 (53.5) | 0.071 | 19 (65.5) | 94 (59.5) | 0.542 |
| Body mass index [kg/m²] | 27.0 (23.9; 32.0) | 27.6 (24.7; 30.7) | 0.789 | 26.1 (22.0; 31.0) | 27.3 (24.2; 31.2) | 0.242 |
| STS-PROM [%] | 9.2 (6.2; 19.1) | 8.0 (4.9; 13.0) | 0.368 | 6.2 (4.6; 12.1) | 6.3 (4.3; 10.0) | 0.665 |
| NYHA III/IV, n (%) | 11 (78.6) | 115 (74.2) | 1.000 | 25 (86.2) | 131 (82.9) | 0.791 |
| Coronary artery disease, n (%) | 8 (57.1) | 84 (54.2) | 0.832 | 16 (55.2) | 91 (57.6) | 0.808 |
| Previous myocardial infarction, n (%) | 4 (28.6) | 18 (11.6) | 0.089 | 2 (6.9) | 17 (10.8) | 0.743 |
| Previous PCI, n (%) | 7 (50.0) | 30 (19.4) | 0.015 | 7 (24.1) | 28 (17.7) | 0.415 |
| Previous CABG, n (%) | 4 (28.6) | 54 (34.8) | 0.774 | 6 (20.7) | 60 (38.0) | 0.073 |
| Atrial fibrillation, n (%) | 5 (35.7) | 56 (36.1) | 0.975 | 9 (31.0) | 70 (44.3) | 0.184 |
| Hypertension, n (%) | 14 (100) | 149 (96.1) | 1.000 | 26 (89.7) | 151 (95.6) | 0.189 |
| Diabetes mellitus, n (%) | 6 (42.9) | 57 (36.8) | 0.652 | 10 (34.5) | 57 (36.1) | 0.869 |
| Previous stroke, n (%) | 1 (7.1) | 17 (11.0) | 1.000 | 8 (27.6) | 19 (12.0) | 0.042 |
| PAD, n (%) | 3 (21.4) | 26 (16.8) | 0.711 | 5 (17.2) | 28 (17.7) | 0.950 |
| Carotid stenosis, n (%) | 3 (21.4) | 23 (14.9) | 0.457 | 6 (20.7) | 28 (18.4) | 0.767 |
| COPD, n (%) | 5 (35.7) | 60 (39.0) | 0.811 | 3 (10.3) | 49 (31.2) | 0.021 |
| CKD >3b, n (%) | 5 (35.7) | 54 (34.8) | 1.000 | 8 (27.6) | 58 (36.7) | 0.345 |
| Immuncompromised status, n (%) | 2 (14.3) | 13 (8.4) | 0.359 | 1 (3.4) | 5 (3.2) | 1.000 |
| Pacemaker prior to ViV, n (%) | 4 (28.6) | 26 (16.8) | 0.277 | 5 (17.2) | 33 (20.9) | 0.654 |
| Left Ventricular Ejection Fraction [%] | 55 (48; 56) | 56 (50; 65) | 0.207 | 50 (44; 60) | 55 (46; 60) | 0.130 |
| Mean gradient [mmHg] | 37 (24; 56) | 38 (31; 48) | 0.661 | 29 (12; 42) | 34 (26; 43) | 0.035 |
| Time aortic valve replacement to ViV [years] | 8.2 (4.9; 13.1) | 8.4 (5.6; 10.9) | 0.896 | 11.5 (9.5; 13.2) | 9.6 (6.9; 12.9) | 0.011 |
| Mode of Failure |  |  | 0.003 |  |  | 0.001 |
| Aortic stenosis, n (%) | 9 (64.3) | 93 (60.0) |  | 8 (27.6) | 84 (53.2) |  |
| Aortic regurgitation, n (%) | 4 (28.6) | 9 (5.8) |  | 10 (34.5) | 16 (10.1) |  |
| Mixed (Aortic stenosis + regurgitation ≥2), n (%) | 1 (7.1) | 53 (34.2) |  | 11 (37.9) | 58 (36.7) |  |
| True internal diameter (TID) |  |  | <0.001 |  |  | <0.001 |
| TID <20 mm, n (%) | 0 (0) | 86 (56.6) |  | 0 (0) | 81 (52.9) |  |
| TID 20.0-22.99 mm, n (%) | 1 (9.1) | 49 (32.2) |  | 5 (19.2) | 49 (32.0) |  |
| TID ≥23 mm, n (%) | 10 (90.9) | 17 (11.2) |  | 21 (80.8) | 23 (15.0) |  |
| Pre-existing moderate/severe PPM, n (%) | 4 (44.4) | 79 (57.7) | 0.500 | 3 (12.5) | 85 (57.4) | <0.001 |

Values are n (%) or median (IQR). ViV indicates valve-in-valve; STS-PROM, Society of Thoracic Surgeons Predicted Risk of Mortality; NYHA, New York Heart Association; PCI, percutaneous coronary intervention; CABG, coronary artery bypass graft; PAD, peripheral artery disease; COPD, chronic obstructive pulmonary disease; CKD, chronic kidney disease; TID, true internal diameter; PPM, prosthesis-patient mismatch

**Online Table VIII.** Procedural characteristics and in-hospital hemodynamic outcomes according to valve type and treatment period

|  | **2007 – 2015** | | | **2016-2022** | | |
| --- | --- | --- | --- | --- | --- | --- |
|  | **Stentless valve**  **(n = 14)** | **Stented valve**  **(n = 155)** | **p-value** | **Stentless valve**  **(n = 29)** | **Stented valve**  **(n = 158)** | **p-value** |
| Access |  |  | 0.673 |  |  | 1.000 |
| Transfemoral, n (%) | 12 (85.7) | 137 (88.5) |  | 29 (100) | 157 (99.4) |  |
| Transapical, n (%) | 2 (14.3) | 18 (11.6) |  | 0 (0) | 1 (0.6) |  |
| Implanted THV |  |  | 0.146 |  |  | 1.000 |
| Self-expanding | 12 (85.7) | 101 (65.2) |  | 25 (86.2) | 137 (86.7) |  |
| Balloon-expandable | 2 (14.3) | 54 (34.8) |  | 4 (13.8) | 21 (13.3) |  |
| Specific THV |  |  | 0.195 |  |  | 0.039 |
| CoreValve | 9 (64.3) | 75 (48.4) |  | 1 (3.4) | 0 (0) |  |
| EvolutR | 3 (21.4) | 26 (16.8) |  | 23 (79.3) | 133 (84.3) |  |
| Portico | 0 (0) | 2 (1.3) |  | 0 (0) | 2 (1.3) |  |
| Lotus | 0 (0) | 0 (0) |  | 1 (3.4) | 0 (0) |  |
| Sapien XT | 2 (21.4) | 34 (22.9) |  | 0 (0) | 2 (1.3) |  |
| Sapien 3 | 0 (0) | 18 (11.6) |  | 4 (13.8) | 19 (12.0) |  |
| Label size of new valve, mm | 26 (25; 29) | 23 (23; 26) | <0.001 | 29 (26; 34) | 23 (23; 26) | <0.001 |
| Need for 2nd THV, n (%) | 0 (0) | 3 (1.9) | 1.000 | 0 (0) | 1 (0.6) | 1.000 |
| Predilatation, n (%) | 9 (69.2) | 103 (67.8) | 1.000 | 20 (80.0) | 100 (67.6) | 0.212 |
| Postdilatation, n (%) | 2 (5.4) | 27 (17.8) | 1.000 | 5 (20.0) | 29 (19.6) | 1.000 |
| Bioprosthetic valve fracture, n (%) | n.a. | 0 (0) | n.a. | n.a. | 0 (0) | n.a. |
| Coronary Protection |  |  |  |  |  |  |
| BASILICA, n (%) | 0 (0) | 0 (0) | n.a. | 0 (0) | 0 (0) | n.a. |
| Chimney stenting, n (%) | 0 (0) | 0 (0) | n.a. | 1 (3.4) | 3 (1.9) | 0.493 |
| Coronary obstruction, n (%) | 1 (2.3) | 0 (0) | 1.000 | 0 (0) | 0 (0) | n.a. |
| Echo before discharge |  |  |  |  |  |  |
| Mean gradient [mmHg] | 15 (11; 19)  n=13 | 17 (12; 24)  n=152 | 0.328 | 8 (5; 15)  n=28 | 14 (10; 20)  n=156 | <0.001 |
| Mean gradient ≥20 mmHg, n (%) | 3 (23.1) | 61 (40.1) | 0.226 | 1 (3.6) | 42 (26.9) | 0.006 |
| Moderate aortic regurgitation after ViV*, n (%) | 0 (0) | 3 (2.0) | 1.000 | 3 (10.7) | 5 (3.2) | 0.103 |

Values are n (%) or median (IQR). ViV indicates valve-in-valve; THV, transcatheter heart valve; BASILICA, Bioprosthetic or Native Aortic Scallop Intentional Laceration to Prevent Iatrogenic Coronary Artery Obstruction; n.a., not applicable. * no severe AR occurred.

**Online Table IX.** VARC-3-defined endpoints and complications after ViV-TAVI according to valve type and treatment period

|  | **2007 – 2015** | | | **2016 – 2022** | | |
| --- | --- | --- | --- | --- | --- | --- |
|  | **Stentless valve**  **(n = 14)** | **Stented valve**  **(n = 155)** | **p-value** | **Stentless valve**  **(n = 29)** | **Stented valve**  **(n = 158)** | **p-value** |
| **VARC-3-defined composite endpoints** | | | | | | |
| 30-day mortality, n (%) | 2 (14.3) | 5 (3.2) | 0.105 | 1 (3.4) | 3 (1.9) | 0.493 |
| Technical success (VARC-3), n (%) | 11 (78.6) | 115 (74.2) | 1.000 | 25 (86.2) | 135 (85.4) | 1.000 |
| Device success (VARC-3), n (%) | 9 (64.3) | 72 (47.1) | 0.217 | 20 (69.0) | 97 (61.4) | 0.439 |
| Early safety (VARC-3), n (%) | 9 (64.3) | 97 (62.6) | 0.899 | 23 (79.3) | 111 (70.3) | 0.320 |
| **VARC-3-defined complications** | | | | | | |
| VARC-3 myocardial infarction, n (%) | 2 (14.3) | 5 (3.2) | 0.105 | 0 (0) | 1 (0.6) | 1.000 |
| VARC-3 Stroke, n (%) | 0 (0) | 7 (4.5) | 1.000 | 0 (0) | 7 (4.4) | 0.598 |
| Major, n (%) | 0 (0) | 4 (2.6) | 1.000 | 0 (0) | 7 (4.4) | 0.598 |
| Minor, n (%) | 0 (0) | 3 (1.9) | 1.000 | 0 (0) | 0 (0) | n.a. |
| VARC-3 Bleeding, n (%) | 1 (7.1) | 37 (23.9) | 0.196 | 5 (17.2) | 17 (10.8) | 0.347 |
| Type 1, n (%) | 0 (0) | 15 (9.7) | 0.617 | 3 (10.3) | 3 (1.9) | 0.049 |
| Type 2, n (%) | 0 (0) | 13 (8.4) | 0.604 | 0 (0) | 7 (4.4) | 0.598 |
| Type 3, n (%) | 1 (7.1) | 7 (4.5) | 0.507 | 2 (6.9) | 7 (4.4) | 0.632 |
| Type 4, n (%) | 0 (0) | 2 (1.3) | 1.000 | 0 (0) | 0 (0) | n.a. |
| VARC-3 Kidney, n (%) | 3 (21.4) | 15 (9.7) | 0.174 | 2 (6.9) | 8 (5.1) | 0.655 |
| Stage 1, n (%) | 1 (7.1) | 8 (5.2) | 0.550 | 2 (6.9) | 4 (2.5) | 0.234 |
| Stage 2, n (%) | 0 (0) | 4 (2.6) | 1.000 | 0 (0) | 1 (0.6) | 1.000 |
| Stage 3, n (%) | 1 (7.1) | 2 (1.3) | 0.230 | 0 (0) | 0 (0) | n.a. |
| Stage 4, n (%) | 1 (7.1) | 1 (0.6) | 0.159 | 0 (0) | 3 (1.9) | 1.000 |
| VARC-3 Access site, n (%) | 2 (14.3) | 36 (23.2) | 0.738 | 3 (10.3) | 22 (13.9) | 0.771 |
| Major, n (%) | 1 (7.1) | 16 (10.3) | 1.000 | 0 (0) | 4 (2.5) | 1.000 |
| Minor, n (%) | 1 (7.1) | 20 (12.9) | 1.000 | 3 (10.3) | 18 (11.5) | 1.000 |
| Need for new pacemaker, n (%) | 1 (10.4) | 14 (10.9) | 1.000 | 1 (4.2) | 10 (8.0) | 1.000 |

Values are n (%). VARC indicates Valve Academic Research Consortium; n.a., not applicable.

**Online Figure 1.** Kaplan-Meier estimates of mortality in patients undergoing valve-in-valve transcatheter aortic valve implantation (ViV-TAVI) into stentless (SL) compared with stented (ST) failed aortic bioprostheses according to treatment period.

**2007 – 2015 2016 – 2022**


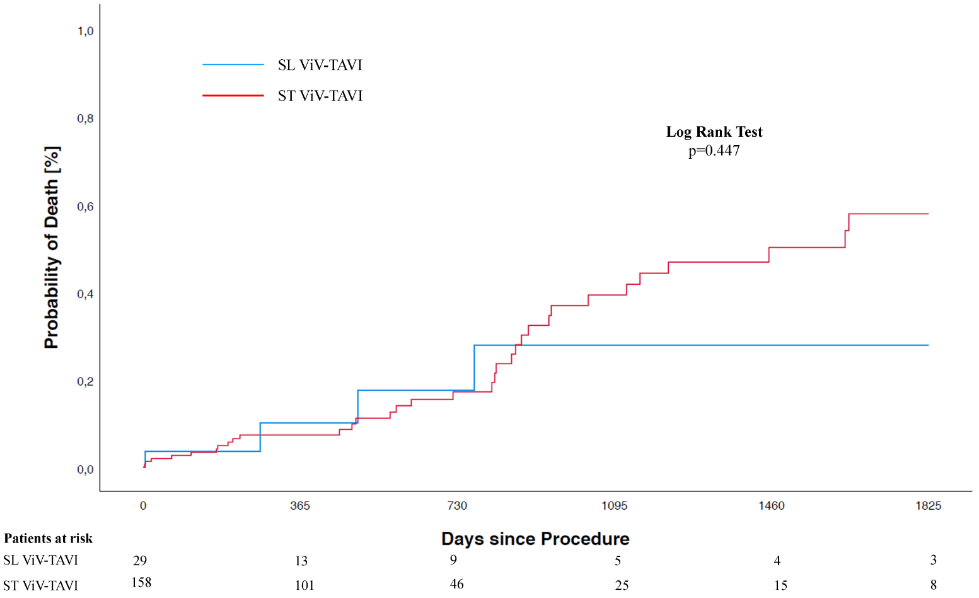

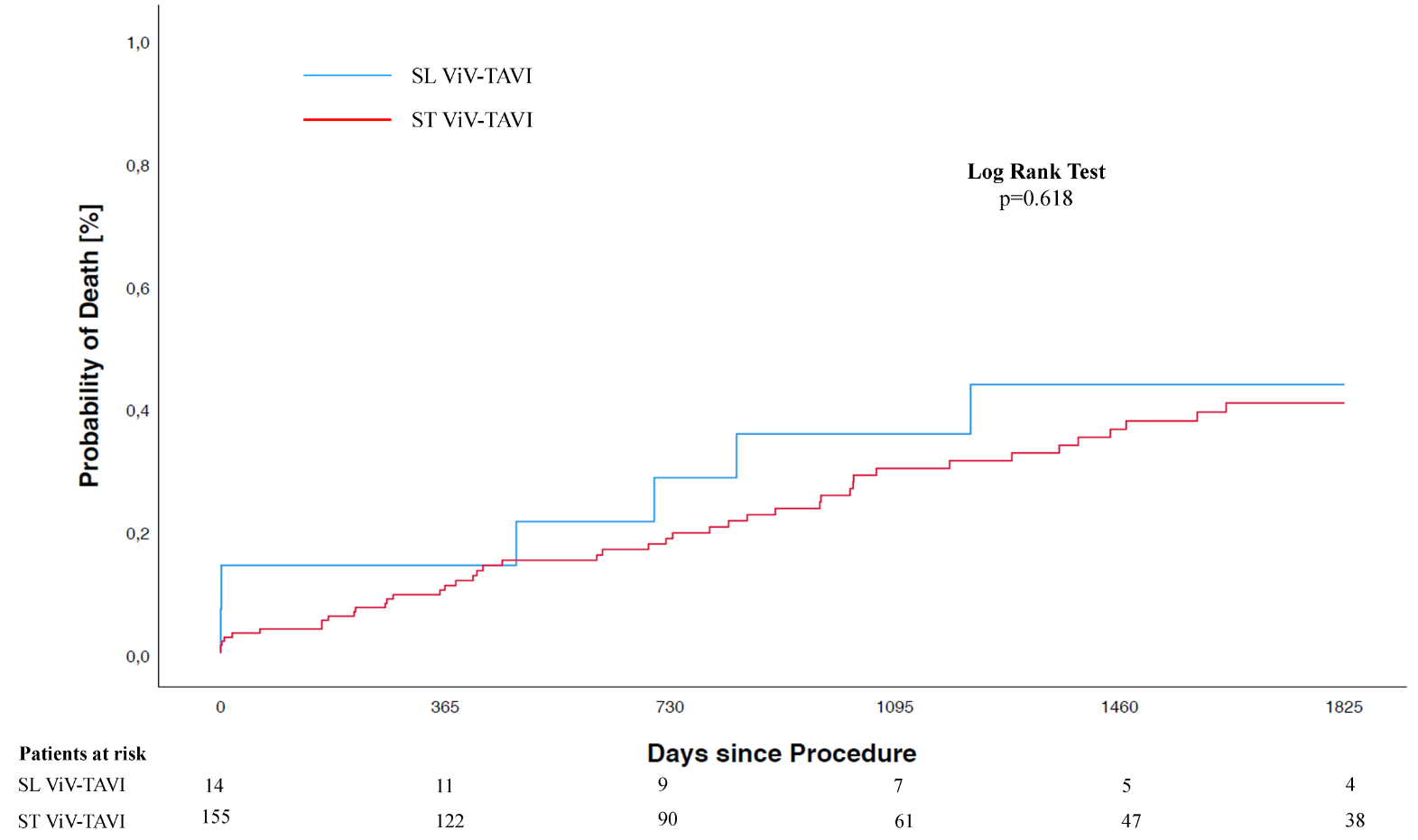

Supplement: Supplementary file 1 — Supplementary file1 (DOCX 203 KB) [file 392_2023_2347_MOESM1_ESM.docx]
